# Dataset of gene cloning and gel filtration chromatography of R-est6

**DOI:** 10.1016/j.dib.2016.04.034

**Published:** 2016-04-21

**Authors:** Surabhi Soni, Annamma A. Odaneth, Arvind M. Lali, Sanjeev K. Chandrayan

**Affiliations:** a DBT-ICT Centre for Energy Biosciences, Institute of Chemical Technology, Nathalal Parikh Marg, Matunga (East), Mumbai 400019, Maharashtra, India; b Department of Chemical Engineering, Institute of Chemical Technology, Nathalal Parekh Marg, Matunga (East), Mumbai 400019, Maharashtra, India

## Abstract

The data presented in this article are connected to the research article entitled “Expression, purification and biochemical characterization of a family 6 carboxylesterase from *Methylococcus capsulatus (bath)*” (Soni et al., 2016 [1]). The family 6 carboxylesterases are the smallest and display broad substrate specificity. The 1 kb gene encoding, a family 6 carboxylesterase – R-est6, was amplified from the genome of *M. capsulatus* (bath strain), and showed in the agarose gel. The corresponding purified protein, after overexpression in *Escherichia coli*, was biochemically studied in the research article (Soni et al., 2016 [1]). R-est6 has hydrophobic patches on the surface so, it is expected to show oligimeric forms. Here, we have confirmed the presence of oligomers by gel filtration chromatography data and the proteins belonging to the different peaks are shown on a SDS-PAGE.

**Specifications table**

**Table:** 

Subject area	Biology
More specific subject area	Molecular biology and biophysics
Type of data	Figures
How data was acquired	The PCR machine (Fig. 1) and The AKTA purification system (Fig. 2)
Data format	Analyzed data
Experimental factors	Cloned PCR product was cloned and expressed in *E. coli* and protein was purified by Ni-NTA affinity column.
Experimental features	R-est6 was affinity purified before loading on superdex 75 column.
Data source location	DBT-CEB-ICT, Mumbai, India
Data accessibility	Data is within this article

## Value of the data

• Confirms the genetic identity of R-est6 in the genome, hence this reported gene could be used as a template in protein engineering experiments.
• Gel filtration chromatography establishes the oligomeric quaternary state of R-est6.
• The reported oligmeric state and buffer conditions would be useful for optimization of the reaction conditions for the immobilization of R-est6.

## 1 Data

**Fig. 1:**
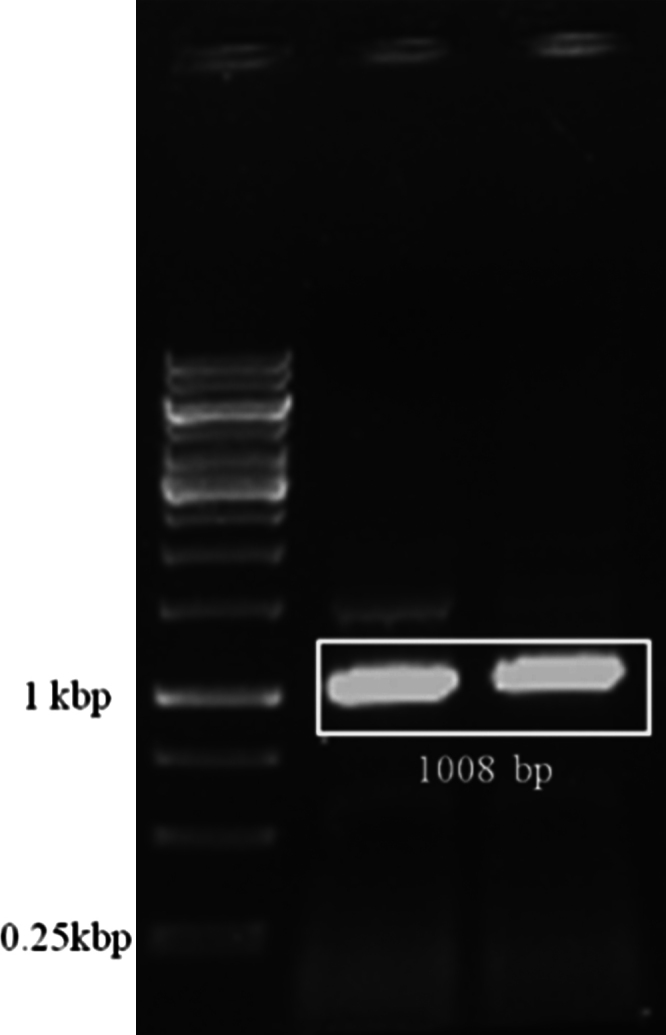
PCR of R-est6 gene, MCA0075 from *Methylococcus capsulatus* (bath strain, ATCC 33009). PCR product of 1008 base pairs is marked in white box with labeled 1 kb ladder in left lane.

**Fig. 2:**
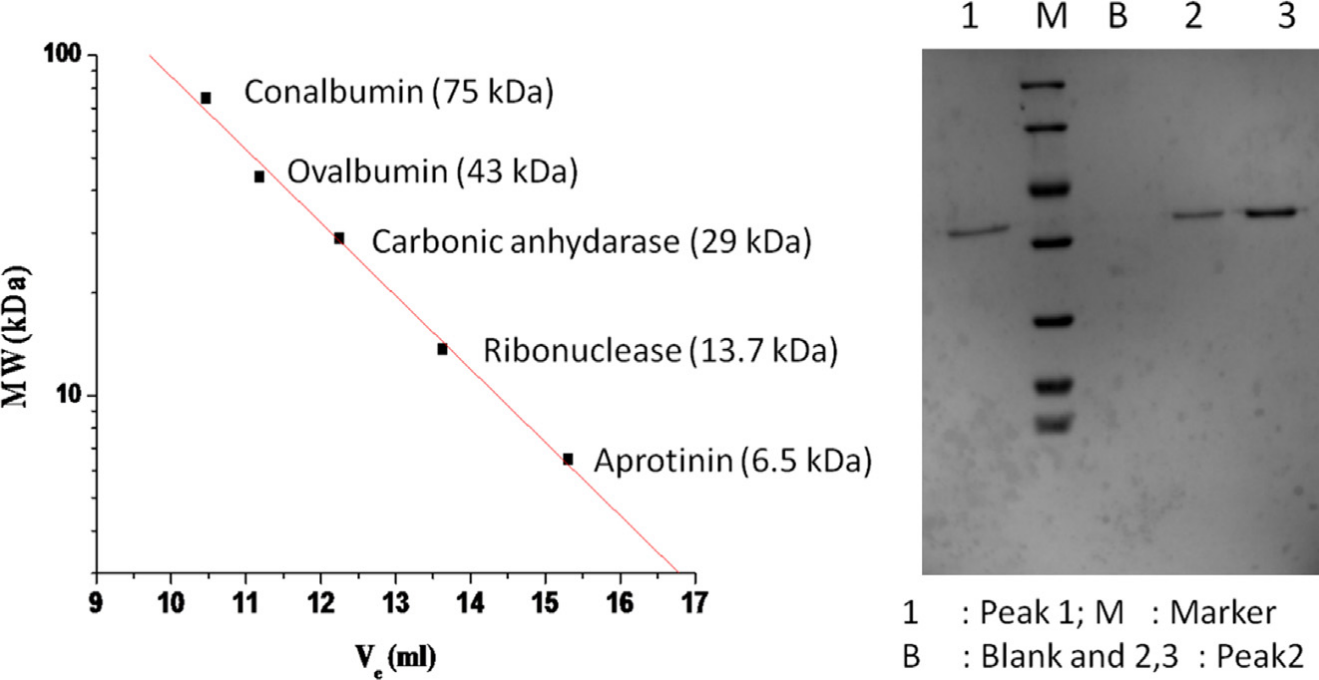
(A) Calibration profile of Superdex 75 Column with gel filtration protein standards of different size as labeled in the plot. (B) Peak 1 and Peak 2 from superdex 75 run, as shown in figure 3C, was confirmed for the presence of R-est6 on a SDS-PAGE. Lane marked 1 corresponds to peak 1 and lane marked 2 and 3 correspond to Peak 2. Molecular weight markers of different sizes are shown in lane marked M and for lane marked B is without any protein loading.

In Fig. 1, PCR products after amplification were separated on agarose gel and were compared against 1 kb ladder to confirm the gene size of R-est6. In Fig. 2, gel filtration chromatogram of R-est6 established the oligomeric state of the purified protein and the molecular weight was ascertained by SDS-PAGE.

## 2 Experimental design, materials and methods

### 2.1 PCR amplification of R-est6

The gene encoding R-est6 (NCBI Reference Sequence WP_010959447.1) from *Methylococcus capsulatus* (ATCC33009D-5) was PCR amplified by using PrimeSTAR HS DNA polymerase (DSS Takara Bio India Pvt. Ltd., New Delhi, India) with following primers: (1) forward primer 5′-ATATTATATTcatatgTCGACATTCGATCGAGGATTCGCGCGGC-3′ and (2) reverse primer 5′-TATATATTATctcgagCTAGCCCTCGACGATAGGGAAATCACTGACC-3′ (restriction sites are in small case). The obtained product was analyzed by 0.8% agarose gel to estimate the size by running the 1 kb ladder.

### 2.2 Size exclusion chromatography of R-est6

The purified R-est6 was analyzed on superdex-75 column that was calibrated with known molecular weight markers to estimate the oligomeric state of the protein [1]. Presence of R-est6 in different peaks was confirmed on SDS-PAGE.
