# Supplementary material for: Dataset of gene cloning and gel filtration chromatography of R-est6
Source: Data Brief. 2016 Apr 21;7:1594–7. doi: 10.1016/j.dib.2016.04.034 (PMC4865677; doi:10.1016/j.dib.2016.04.034)
Supplement: Supplementary file 1 — Supplementary material [file mmc1.docx]

Conflict of interest: None
